# Efficacy and safety of ShenSongYangXin Capsule combined with antiarrhythmic drugs for atrial fibrillation: A protocol for systematic review and network meta-analysis

**DOI:** 10.1097/MD.0000000000022367

**Published:** 2020-09-18

**Authors:** Shuqing Shi, Jingjing Shi, Qiulei Jia, Shuai Shi, Guozhen Yuan, Yuanhui Hu

**Affiliations:** a Cardiovascular Department, Guang’anmen Hospital, China Academy of Chinese Medical Sciences; b Graduate School, Beijing University of Chinese Medicine, Beijing, China.

**Keywords:** antiarrhythmic drugs, atrial fibrillation, network meta-analysis, protocol, randomized controlled trials, shen-song-yang-xin capsule

## Abstract

**Background:**

Shen-Song-Yang-Xin Capsule (SSYX), a Chinese patent medicine, combined with antiarrhythmic drugs (AADs) in the treatment of atrial fibrillation (AF) has been widely applied in clinical practice, but the results are controversial. This study aims to conduct a network meta-analysis (NMA) based on data from the randomized controlled trials (RCTs) to evaluate the efficacy and safety of SSYX combined with ADDs in the treatment of AF.

**Methods and analysis:**

A comprehensive systematic literature search will be conducted in Cochrane Library, PubMed, Web of Science, EMBASE, Chinese Biomedical Literature Database (SinoMed), Chinese National Knowledge Infrastructure (CNKI), and WanFang database for RCTs about SSYX combined with ADDs. The primary outcomes will be the frequency of AF attack and P-wave dispersion, and the secondary outcomes will be the symptom improvements, left atrial diameter, and adverse events. Statistical analyses will be conducted by using WinBUGS software (version 1.4.3), Stata software (version 14.0) and RevMan software (version 5.3).

**Results:**

The results of this NMA will provide a high-quality evidence for the efficacy of SSYX combined with ADDs in the treatment of AF, and a ranking of the therapeutic classes will also be presented.

**Conclusion:**

The protocol will provide updated evidence for the application of SSYX for AF.

**International Platform of Registered Systematic Review and Meta-Analysis Protocols (INPLASY) registration number:**

The protocol of this systematic review and meta-analysis was registered on the INPLASY website (https://inplasy.com/inplasy-2020-8-0075/) and INPLASY registration number is INPLASY202080075.

## 1 Introduction

Atrial fibrillation (AF) is the most common clinical arrhythmia characterized by the high-frequency electrical activity of the atrium and unsynchronized atrial contraction.^[1]^ With a global prevalence of 33.5 million, the number of AF patients is continuously increasing and is estimated to rise to 12.1 million in the United States by 2030.^[2,3]^ The proportion of adults over 40 years old suffering from AF in developed countries can reach 1 quarter.^[4]^ The high incidence of AF is associated with increased rates of mortality and disability. The risk of stroke is 4 to 5 times higher in AF patients than non-AF ones,^[5]^ which leads to a nearly 20% mortality rate and nearly 60% disability rate.^[6]^ One percent of the annual medical expenditure in the UK and up to $26 billion in US medical expenditure are related to AF, most of which is hospitalization costs.^[7,8]^ AF has become a major public health problem that hinders the improvement of national health and social development.

In recent years, significant progress has been made in the clinical treatment of AF. New oral anticoagulant drugs and vitamin K antagonists can remarkably reduce the risk of stroke and death in patients with AF.^[9,10]^ In addition, heart rate control^[11]^ and heart rhythm control^[12]^ can effectively improve AF-related symptoms and cardiac function. For paroxysmal AF patients who have failed to respond to drug therapy, the guidelines recommend radio frequency ablation. But for persistent AF and other types, the success rate of postoperative sinus rhythm recovery is not high, and the long-term follow-up results are not satisfactory. Surgical AF ablation such as maze surgery is mostly used for AF patients undergoing cardiac surgery simultaneously, but patients with simple AF have a low acceptance of it.

The results of many clinical randomized controlled trials (RCTs) have indicated that complementary and alternative medicine has similar antiarrhythmic effects to AADs and has fewer adverse events.^[13–15]^ As a part of it, Chinese medicine has been used to prevent and treat arrhythmia in China for thousands of years. Shen-song-yang-xin Capsule (SSYX) is a traditional Chinese patent medicine, it is composed of 12 kinds of Chinese herbal medicines, including *Ginseng* (ren shen), *Radix Ophiopogonis*(mai dong), *Fructus Corni* (shan zhu yu), *Salvia Miltiorrhiza* (dan shen), *Semen Ziziphi Spinosae* (suan zao ren), *Herba Taxilli* (sang ji sheng), *Nardostachys Chinensis* (gan song). The formula of SSYX is based on the traditional Chinese medicine (TCM) theories of tonifying Qi, nourishing Yin, promoting blood circulation to remove the obstructions to calm the mind. It has been widely used in the treatment of patients with various types of AF, and proved by a large number of evidence-based medicine that SSYX has a significant effect and can greatly reduce the side effects that may occur by using the AADs.

In the past, although some RCTs have compared the clinical efficacy of SSYX combined with AADs, but there were no studies comparing different combinations. In addition, although many positive outcomes have been demonstrated, however, the optimal threshold, the treatment regimens, and the efficacy and safety of using SSYX combined with AADs remain to be determined. Therefore, we are going to conduct this network meta-analysis (NMA) to confirm the clinical efficacy and safety of SSYX combined with AADs, to determine the optimal threshold and treatment regimen, and to provide evidence that individualized TCM therapy is beneficial in the treatment of AF.

## 2 Material and methods

### 2.1 Study registration

This meta-analysis will be performed according to the Preferred Reporting Items for Systematic Reviews and Meta-Analyses (PRISMA-NMA) statement.^[16]^ The protocol of this systematic review and meta-analysis was registered on the INPLASY website (https://inplasy.com/inplasy-2020-8-0075/) and INPLASY registration number is INPLASY202080075.

### 2.2 Data sources and search strategy

In this study, we will search 7 commonly used databases: Cochrane Library, PubMed, Web of Science, EMBASE, Chinese Biomedical Literature Database (SinoMed), CNKI, and WanFang database. The retrieval time begins on the database's build date and ends in December 2019. The references of included studies will be tracked and those meeting the inclusion criteria will be supplemented. The search strategy will be adjusted according to the characteristics of different databases. Table 1 shows the details of the search strategy in PubMed.

**Table 1:**
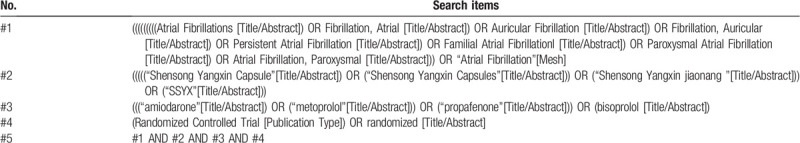
PubMed search strategy.

### 2.3 Inclusion and exclusion criteria

#### 2.3.1 Types of studies

RCTs regarding SSYX combined with AADs for AF will be included without language restriction.

#### 2.3.2 Types of participants

All adult patients diagnosed with AF (aged 18 and over, with no upper age limit) will be included in the study. We will follow the 2016 European Society of Cardiology preliminary criteria for the clinical diagnosis of AF^[17]^ and the AHA/ACC/HRS 2019 guidelines for the management of patients with AF.^[18]^

#### 2.3.3 Types of interventions

Both the experimental group and the control group routinely treated for basic cardiovascular diseases, including antihypertensive, anticoagulant, lipid-lowering, diuretic and other treatments. According to the literature, the intervention methods were designed into following groups: SSYX with Amiodarone; SSYX with Propafenone; SSYX with Metoprolol; SSYX with Bisoprolol; others: alternative AADs.

#### 2.3.4 Types of comparison

Patients in the control group are those who only have received AADs treatment without SSYX.

#### 2.3.5 Types of outcomes

The primary outcomes will be the frequency of AF attack and P-wave dispersion, which will be measured by electrocardiogram or 24 hours dynamic electrocardiogram result.

The secondary outcomes will be: Symptom improvement, including palpitations, chest tightness, dizziness, blackness, fatigue, and other symptoms; left atrial diameter that measured by echocardiography; adverse events.

### 2.4 Selection of studies

**Figure 1:**
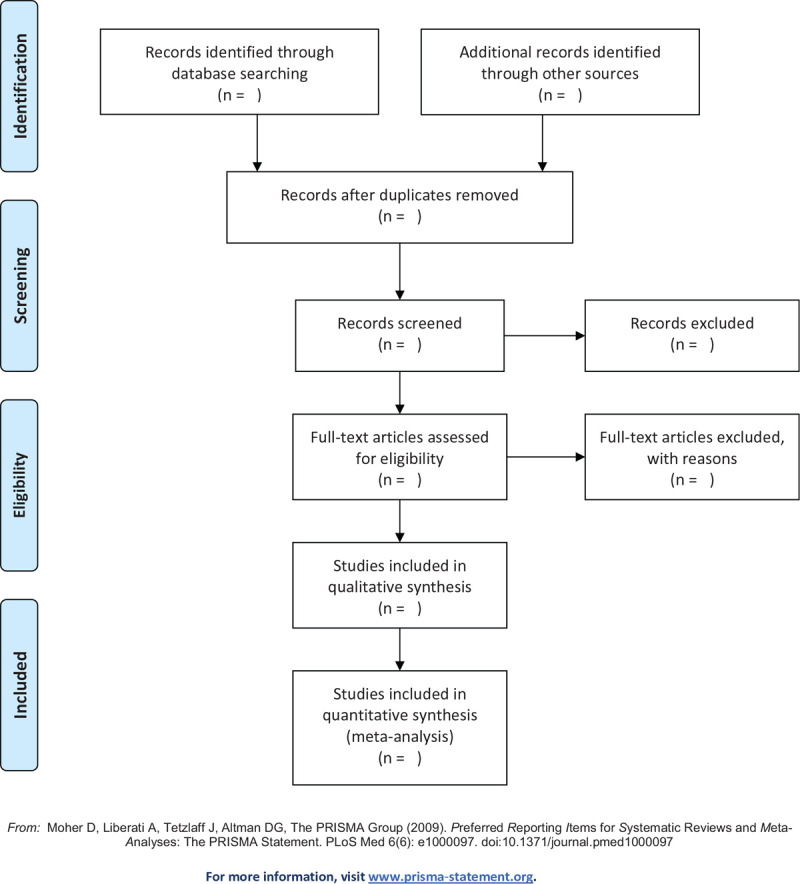
Flow diagram of studies search and selection.

Three reviewers (SS, QLJ, and JS) will independently select the studies according to the inclusion criteria, then removing the duplicate and unqualified articles by reading the titles as well as abstracts. For the articles that meet the inclusion criteria, we will read the full texts to determine their eligibility. In addition, the reference list of eligible articles will be manually retrieved. If there is any inconsistency between the 2 reviewers, the corresponding author (YH) will make the final decision. The reviewers participating in the search are experienced clinical AF experts or evidence-based medicine methodology experts. The literature retrieval process of this study will use the PRISMA flowchart, as shown in Figure 1. For each excluded study, reasons for exclusion will be given.

### 2.5 Data extraction and management

The 2 review authors (SS and GY) will independently extract the data of the included studies according to the data extraction table. The extracted information includes: author, year of publication, type of study, sample size, and follow-up time. If the data that needs to be extracted is not available in full text, we will contact the corresponding author. The multiarm test will be split into a double-arm test to ensure that the results can be combined.^[19]^

### 2.6 Risk of bias assessment

The quality of the included studies will be assessed according to the Cochrane collaboration's risk of bias tool.^[20]^ The evaluation contents include random sequence generation, allocation concealment, participants and personnel blindness, outcome evaluation blindness, incomplete outcome data, and selective reports. The risk of bias will be classified as “high risk,” “low risk,” and “unclear risk.”^[20]^

### 2.7 Data synthesis and analysis

#### 2.7.1 Standard pairwise meta-analysis

Standard pairwise meta-analysis will be performed using Stata V.14.0 software. Odds ratio (OR) will be used as the effect size for enumeration data, mean difference will be used for measurement data, and 95% confidence interval (CI) will be used for interval estimation. To explore between-study variability, the Cochrane Q statistic with the χ^2^ test and the Higgins I^2^ test for heterogeneity will be used.

#### 2.7.2 Network meta-analysis and network geometry

WinBUGS 1.4.3 will be used for network statistical analysis,^[21]^ in which the posterior parameters will be calculated by Markov chain Monte Carlo methods. After an initial burn-in of 50,000, we will operate another 100,000 iterations.^[22]^ Surface under the cumulative ranking probabilities (SUCRA) values will be applied to rank the examined treatments, with SUCRA values of 100% and 0% assigned to the best and worst treatments, respectively.^[23]^

The network geometry will be drawn by Stata 14.0. The larger arm indicates a larger amount of basic data of the intervention, and the larger circle area indicates a better effectiveness of the intervention.^[24,25]^ Besides, a comparison-adjusted funnel plot was used to test for the publication bias.

#### 2.7.3 Assessment of transitivity and inconsistency

Transitivity is the fundamental assumption of indirect comparisons and network meta-analysis, and its violation threatens the validity of the findings obtained from a network of studies.^[26]^ We will use local and global methods to assess whether there is statistical inconsistency in the data.^[27]^ Specifically, we will use the loop-specific approach^[28]^ to detect loops of evidence that might present important inconsistency as well as the node splitting approach^[29]^ to detect comparisons for which direct estimates disagree with indirect evidence from the entire network.

#### 2.7.4 Subgroup and sensitivity analyses

To assess the impact of covariates and areas (e.g., gender distribution, severity of illness graded) of heterogeneity in our sample, we will explore subgroup analysis and/or use meta regression.

We will conduct a sensitivity analysis to exclude each study sequentially and combine the remaining studies to identify the impact of each study on the overall outcome.

#### 2.7.5 Assessment of publication biases

Publication bias will be assessed using funnel plots and the Egger test for direct comparisons with 4 or more studies. In the funnel plots, if the points representing the included studies are evenly distributed, it means that the publication bias is small.

#### 2.7.6 Ethics and dissemination

It is not necessary for ethical approval because it is based on published studies. The protocol will be disseminated in a peer-reviewed journal or presented at a topic-related conference.

## 3 Discussion

AF, as an arrhythmia disease with high prevalence, high disability rate, and high mortality rate, however, has poor clinical treatment effect. This network meta-analysis will conduct a detailed summary and analysis of SSYX combined with AADs in the treatment of atrial fibrillation to find the best treatment plan. Although the comparison of therapies in a network meta-analysis is conducted based on the direct comparisons of treatments and the indirect comparisons based on a common measurement, some factors may affect the outcomes of the analysis such as the number of articles included in the network, so we use the heterogeneity test, the inconsistency test, and several approaches to deal with those factors.^[30,31]^

However, there are still limitations in this network meta-analysis. Some studies of low quality may decrease the significance of the results in network meta-analysis. Moreover, heterogeneity of different studies may affect the final results of this study. Despite the limitations, a systematic review with the newest data should be conducted to show such combination of therapeutic method with better atria conduction and quality of life. Therefore, it is worthy to display this protocol for a systematic review. We hope our work will provide more convincing evidence to the clinicians about the treatments for patients with AF.

## Acknowledgment

The authors acknowledged Mr Kang Ren for his contribution to edit the English text of a draft of this manuscript.

## Author contributions

**Conceptualization:** Shuqing Shi, Qiu lei Jia.

**Data curation:** Shuqing Shi, Jingjing Shi

**Methodology:** Shuai Shi

**Project administration:** Yuanhui Hu

**Writing – original draft:** Shuqing Shi.

**Writing – review & editing:** Qiu lei Jia, Guozhen Yuan.

## Abbreviations

Abbreviations: AADs = antiarrhythmic drugs, AF = atrial fibrillation, CNKI = Chinese National Knowledge Infrastructure, NMA = network meta-analysis, OR = odd ratio, RCTs = randomized controlled trials, SSYX = Shen-song-yang-xin Capsule, TCM = traditional Chinese medicine.
